# Meeting the needs of a complex population: a functional health- and patient-centered approach to managing multimorbidity

**DOI:** 10.15256/joc.2016.6.83

**Published:** 2016-08-24

**Authors:** Tara Sampalli, Robert Dickson, Jill Hayden, Lynn Edwards, Arun Salunkhe

**Affiliations:** ^1^Integrated Chronic Care Service, Primary Health Care, Nova Scotia Health Authority, Nova Scotia, Canada; ^2^Primary Health Care, Nova Scotia Health Authority, Nova Scotia, Canada; ^3^Department of Medical Informatics, Dalhousie University, Nova Scotia, Canada; ^4^Department of Community Health and Epidemiology, Dalhousie University, Nova Scotia, Canada

**Keywords:** multimorbidity, functional health approach, patient engagement, chronic disease management, patient-centered

## Abstract

Individuals with multimorbidity have complex care needs along with significant impacts to their functional health and quality of life. Recent evidence-based and experience-based explorations have revealed the importance of patient perspectives and functional health management in improving care delivery and health outcomes for individuals with multimorbidity. The impact of managing multimorbidity is evident at multiple levels of healthcare – the individual, the provider, and the system. Our local experience dealing with these challenges has led to the development of a functional health model that includes patient perspectives in care delivery within the Integrated Chronic Care Service (ICCS) of the health authority in Nova Scotia. In this paper, we present a discussion of the challenges, guiding models, and service-level transformations that have been integrated into care delivery at the ICCS to meet the healthcare needs of people with multiple health conditions. We describe our redesign strategies for care team planning, treatment approach, and patient inclusion.

## Introduction

People with chronic conditions require a range of health services delivered by primary care, community care, as well as acute care professionals and specialists. Navigating these services can be complex and, consequently, patients may fall between the gaps in care handovers [1]. Individuals with multimorbidity (that is, having more than three chronic conditions) are particularly challenging as they require timely, relevant, and coordinated care. Multimorbidity affects more than one in four people in Canada, and the number of patients with multimorbidity seen in primary care is increasing [2, 3]. Understanding the impact of multimorbidity at all important healthcare levels, namely, the individual, the provider, and the health system is not only a challenge but also presents an opportunity. In order to create the right impact at all these levels, a system-level redesign is essential [4–8].

Recent experiences have shown that patient perspectives of care are an important consideration and should provide guidance to understand the complexity and severity of problems that individuals with multimorbidity experience in their daily lives [7, 8]. Specifically, including patient perspectives in the care delivery process is necessary since the support and care required by people with multiple chronic conditions can vary, based on several factors in addition to the disease count [9–11]. Two patients with the same type and number of comorbidities may vary in their perceptions of wellness and in their experiences of their illness. Additionally, other factors that could be important in their care and support may include socio-economic and environmental factors, emotional and psychosocial distress, existential and spiritual distress, ethnicity and cultural factors, social and health system supports, and other lifestyle factors [10, 12]. At the health system level, the challenges experienced by individuals with multimorbidity are highlighted by the current limitations of the system that is primarily designed around acute care and single condition focus. Multimorbidity can pose significant challenges to the system in terms of timeliness, relevance, effectiveness, and efficiency; a redesign to shift focus from a single condition to a more comprehensive view of the person is needed. Community resources and supports are similarly not well established to meet the needs of individuals with complex diseases who may benefit from upstream activities that support health promotion and early prevention. Primary care providers and specialists experience challenges due to lack of education related to multimorbidity (beyond disease counts), awareness of relevant resources, integrated information systems, and care management systems. Consequently, these patient-level challenges arise from the limitations in the system and provider-level preparedness to meet the complex needs of this population. Risk factors of chronic disease, such as obesity, can also add to the complexities of disease management [10, 13], and functional health appears to be an important disabling factor for these individuals [10, 14, 15].

In this paper, we discuss the innovations and transformations that have been implemented in a clinic within a health authority in Nova Scotia to address many of the challenges and opportunities identified at patient, program, and system levels for people with multiple chronic conditions. Specifically, some of the transformations are around the inclusion of patient perspectives in care delivery and a functional health approach in the management of multimorbidity.

## Multimorbidity experience and innovations in a treatment facility in Nova Scotia

The Integrated Chronic Care Service^1^ (ICCS) is part of Primary Health Care^2^ (PHC) in the provincial health authority in Nova Scotia, and offers integrated care for individuals who have challenging and complex diagnoses and medically unexplained conditions. The clinic receives local, national, and international referrals.

PHC in Nova Scotia is a complex system with urban, suburban, and rural service locations, with team-based and individual practices, and various payment plans and support services in the community. PHC is responsible, as a portfolio, for primary care and chronic disease management. Work in PHC is grouped into two broad categories: direct service delivery, and initiatives. The majority of team members work in one of the service delivery areas: Diabetes Management Centres (Urban and Rural), the ICCS, Community Health Teams, Rural Health Teams, prideHealth, Dalhousie Family Medicine clinics, Community Health Centres, the Nova Scotia Brotherhood Initiative, and our Community Health and Wellness teams. Our initiatives are supported by a relatively small number of team members and are typically a launching point for direct clinical service or supporting service: Urgent Care Centres, Collaborative Emergency Centres, Mobile Outreach Street Health, Behaviour Change Institute, Primary Health Care Connections, Your Way to Wellness, Building a Better Tomorrow Together, and others. Following an internal review, it was determined that developing a consistent value-based patient and family engagement strategy was essential to support a strong, effective, and efficient PHC system. The ICCS is an integral part of the PHC system supporting chronic disease and self-management for individuals with complex chronic conditions.

**Figure 1:**
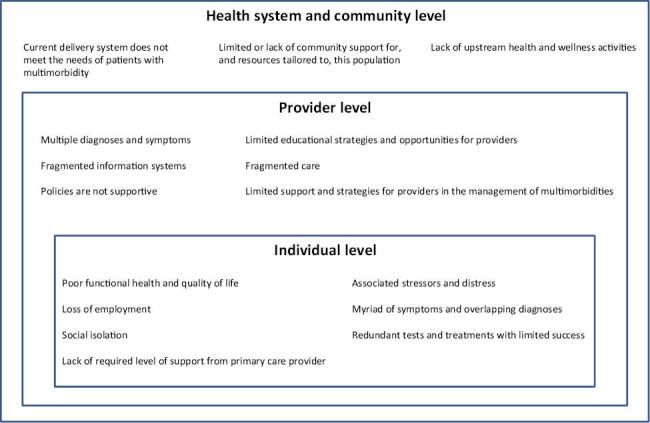
Levels of challenges for the patient population with multimorbidity.

The challenges of managing multimorbidity are a primary focus for this service as over 75% of its patient population has more than three chronic conditions. Some of the challenges experienced at the system, service, and individual levels (Figure 1) have been examined and discussed in detail in our earlier publications, including the evolution of the ICCS into the service it is today [7, 8, 15, 16].

In a recent paper, Sampalli *et al*. [8] discussed in detail system-level redesign considerations, a methodology, and improvements made to enhance care and care experiences for multimorbidity. In this paper, we discuss specific strategies for care delivery at the service and patient levels. We present our experiences realigning a community-based service in primary healthcare to meet the needs of this complex patient population. We discuss strategies for care team redesign, treatment approach, and patient inclusion strategies.

## ICCS care delivery model

### 1. Care team design and work processes to address complex needs of patients

The multidisciplinary team of clinicians at the ICCS works closely with primary care providers in managing the care for such individuals. The multidisciplinary team comprises a care coordinator, a physician, a nurse practitioner, nurses, occupational therapists, a psychologist, a psychotherapist, and a clinical dietitian. The service is part of PHC and funded by the health authority, with all staff having salaried positions to support the needs of the complex population, including the physician, who is a part of the multidisciplinary care team. The standards for service are aligned with the standards of care outlined by PHC in the health authority and by accreditation and chronic disease standards. The care team supports a range of healthcare needs for patients, including medical, dietary, psychosocial, psychological, functional, and rehabilitation needs.

The details of the interventions offered for the population with multimorbidity are described in another paper [14]. In this paper, we only describe elements of redesign for this population related to team functioning, guiding models for the care team, and patient involvement in care.

#### Guiding models and frameworks

**Figure 2:**
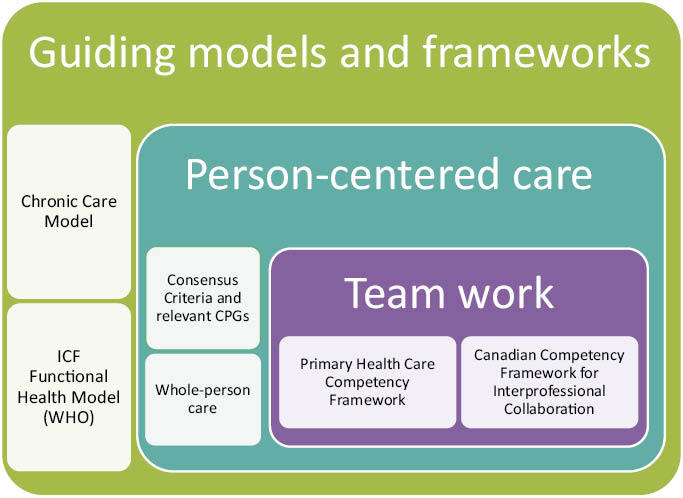
Guiding models and framework for the Integrated Chronic Care Service (ICCS) team. CPG, clinical practice guideline; ICF, International Classification of Functioning, Disability and Health; WHO, World Health Organization.

There are several guiding models and frameworks for the care team to help support the needs of the population it serves, including the Chronic Care Model (CCM) [17] and the World Health Organization’s *International Classification of Functioning, Disability and Health* (ICF) [18] (Figure 2). The CCM is a multipronged approach to delivering integrated care for chronic conditions and has been widely adopted by organizations all over the world. It is one of the guiding models for patients seen at the ICCS who have chronic conditions and require support along all elements outlined in the CCM and the Expanded CCM [18].

The ICF is also a reference model for the care team [19]. This model outlines a standard language and a framework for the description of functional health and health-related states. ICF’s multipurpose classification supports the ICCS care team in meeting the functional health management needs of its patients using the described changes in the classification in body function and structure specified as a person’s ability to function in a standard environment and their current level of performance. This model holds an important role in the ICCS care delivery as patients have significant functional health limitations as seen in their clinical characteristics (average daily activity score of 11.4). The ICCS care team uses ICF as a guiding model to explore all aspects of functional health needs for its patients. Embedded in this strategy is the application of the Canadian Occupational Performance Measure (COPM) [20] as a method to track the progress of patient self-selected functional health goals.

#### Person-centered care

Along with relevant clinical guidelines and diagnostic criteria, the team’s person-centered approach is also grounded in the whole-person model of care for complex populations. This model outlines a coordinated approach that addresses medical, behavioral, psychosocial, and other needs of complex populations [21]. In this approach, care coordination is considered a key element of care delivery, coordinating relevant patient information and action among the multiple healthcare providers, caregivers, such as family members, and community services and employers. At the ICCS, occupational therapists support the care coordination process for patients and for the team.

#### Team work

Finally, the interdisciplinary team’s performance is guided by the Primary Health Care Competency Framework and the Canadian Competency Framework for Interprofessional Collaboration [22]. These frameworks guide the team’s collaborative work, competencies, and functions as they relate to interprofessional collaborations and specifically as outlined for the PHC system.

### 2. Patient’s voice in the care delivery process

**Figure 3:**
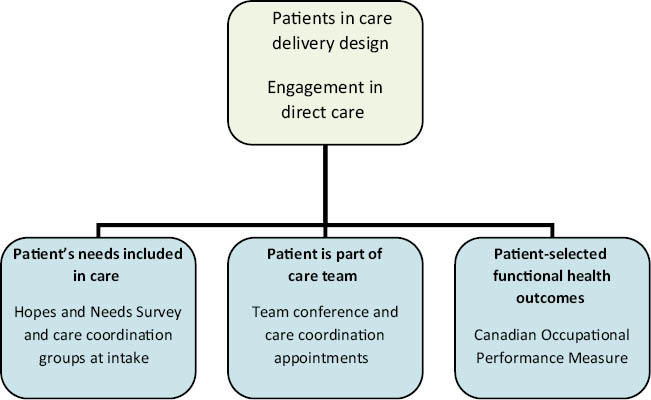
Patient involvement in direct care delivery.

Based on evidence- and experience-based feedback [8, 14], the ICCS team has taken several steps to include patient perspectives and voice in the direct delivery of care (International Association for Public Participation – IAP2 Framework) [23]. Patients are part of the team in the intake and key phases of care delivery, so that they can co-design their care plan that brings value, addressing potential gaps in care, recognizing that a patient with multimorbidity accesses a range of services and multiple care providers (Figure 3). The ICCS care commences with a set of group visits to help patients identify their needs based on gaps in care. The group programs are led by care coordinators. In these group programs (two groups), the care coordinators work closely with new patients to: (i) orient them to the ICCS care delivery process and help new patients explore if the service meets their initial expectations; (ii) identify their health needs and gaps in care (Hopes and Needs survey); (iii) educate patients about the whole-person care model and examine their relevance in the context of multimorbidity; (iv) initiate conversations around self-management, self-care, and functional health management needs and supports; (v) examine needs and supports from primary care providers and community resources; and finally, (vi) discuss next steps to care at the ICCS.

Where to begin care and how to tailor the care needs are important questions for clinicians in the management of care needs for individuals with multiple problems. Comprehensive information is gathered on all new patients to understand the full scope of their care needs as shown in Table 1. The Hopes and Needs survey is another novel element of the care delivery process as a response to these questions and is a simple tool designed by ICCS patients and the care team to allow patients to self-identify their care needs and is a starting point for care management (see Supplementary File). Through this survey, which is administered at several of the initial visits, the patients can choose to engage in a multidisciplinary team approach, a physician-only visit, or opt for specific treatment strategies based on their immediate needs, such as dietary management. This survey is also used as a conversation tool by the care coordinators to engage and support individuals in identifying and co-designing their care needs.

Following intake, patients participate in case conference meetings at key phases of care delivery. These meetings allow patients to connect with their care coordinator and relevant team members to assess their progress, self-management needs, and offer feedback on what is adding value to their care. These meetings also provide patients with an opportunity to assess their follow-up care needs and supports for their primary care provider.

**Figure 4:**
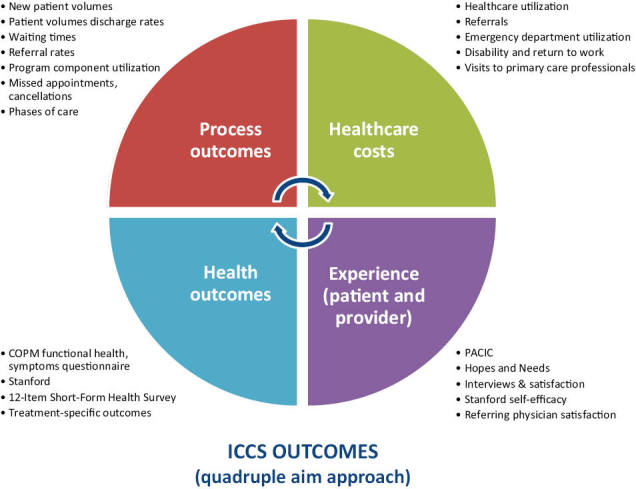
Integrated Chronic Care Service (ICCS) ‘Service’, ‘Patient’ and ‘Provider’ level outcomes. COPM, Canadian Occupational Performance Measure; PACIC, Patient Assessment of Chronic Illness Care.

Based on patients’ feedback, outcomes that are meaningful and relevant at all different levels, namely, patient, provider, and service, have been identified as shown in Figure 4. Over the years, it has become apparent to the ICCS care team and patients that, more important than disease-specific clinical outcomes, are those outcomes that measure the individual and societal consequences of the illness. At the individual level, these measures reflect daily functions, functional goals, confidence in self-management, physical and mental impact of illness and coping, and satisfaction with care received [20, 24, 25]. At the service level, these measures address engagement in care (missed or cancelled appointments), utilization of health services, rehabilitation, and reintegration into community, as relevant [26]. At the service level, the measures also look at the needs of the care team and effectiveness in delivering care. Based on these, the ICCS outcomes have evolved into a comprehensive set of measures in multiple domains as shown in Figure 4. Many of these outcomes are measured using validated and standardized survey instruments as listed in Table 1. The process outcomes, such as waiting times, missed appointments, new patient volumes, and program component utilization are obtained from our administrative systems.

Through a recent initiative in the PHC system in Nova Scotia, a system-level strategy to include patient and family advisors as equal partners and members in Quality Teams for programs and services has been developed to support the design and implementation of meaningful quality improvement activities. An example of this type of role for patients with positive outcomes has been demonstrated in the ICCS service [11].

### 3. Are we making a difference with this new approach?

We have empirically observed that the needs of a person with multiple health conditions go beyond the disease count. Patient characteristics and additional requirements of this population that have been identified in recent literature are consistent with the patient characteristics seen in the ICCS clinic. The ICCS is a provincial service in Nova Scotia that receives referrals from within Nova Scotia, across Canada, and internationally, having over 10,000 patient visits per year with approximately 700 new referrals each year.

Following multiple feedback opportunities with patients, team members, and relevant stakeholders, and with the intention of obtaining a clear and relevant view of the patient’s needs, a comprehensive set of data is gathered on all new patients (Table 1). Information gathered can be one time at intake or pre- to post-intervention, based on the relevance for care planning.

The profile of individuals with multimorbidity seen at the ICCS is discussed in this section to emphasize the multifaceted nature of this problem. The baseline information gathered using the sources listed in Table 1 is discussed in a sample of 456 individuals seen at the ICCS over an 18-month period (from September 2013 to April 2015). The average age of patients seen at the ICCS was 45.8 years and the majority (87%) of patients were female (Table 2). Nearly half of the patients seen were still employed.

The clinical characteristics of patients are shown in Table 3. Over 65% of the patients seen have >5 chronic conditions, with over 65% having musculoskeletal conditions, about 45% having respiratory conditions, and nearly 30% having overlapping mental health symptoms or diagnoses. Related to this, most patients hold a fragmented view of themselves related to specific diagnoses, which makes it challenging to treat the individuals as a whole person [14, 16].

The clinical profile of the population indicates overlapping health concerns, pain and fatigue symptoms in a high percentage of patients, and limitations in ability to perform daily activities and physical capacity. Consequently, the overall perception of health and confidence in self-managing their condition is poor in most patients. Baseline characteristics also show increased usage of the health system (average emergency department visits = 1.07 in 6 months; average visits to primary care provider = 2.65 in 6 months, and average number of providers involved in care, in addition to their primary care provider = 2.43).

Table 4 shows the functional health goals self-selected by our sample of ICCS patients. The top five goals selected by more than 75% of the patient population are reflective of the patient characteristics and previously mentioned challenges for these patients – self-management, productivity, and societal functions. The type of care goals and outcomes selected by the patients are very different from the traditional way of a system-level thinking of goals and outcomes, which are typically centered on clinical outcomes, such as blood glucose levels or blood pressure. Often, the outcomes measured are not even relevant in a clinical care context or in supporting goals for care. Patients with multiple health challenges are thinking about housework, quality time with their family, isolation from societal activities, including their employment and leisure. This emphasizes the importance of working with the patients in developing care that is meaningful and relevant in addressing needs that go beyond disease management or just clinical outcomes.

A pilot initiative with a small sample of patients (*n*=20) following the redesign of the service has shown promising results [14]. Symptom scores showed significant shifts after intervention in terms of overall perception of health and in fatigue scores. The COPM showed statistically significant shifts in the performance and satisfaction in self-selected functional health goals post intervention. The top categories identified included exercise, work, energy levels, housework, and preparation of meals. This initiative is now being explored in a larger sample size and a rigorous study design.

## Discussion and conclusions

Transforming health systems to address the needs of individuals with multimorbidity can be complex and challenging, but it is seen as an essential step. Shifting from a single disease focus and including patient perspectives can play important roles in these transformations. Such transformations can have significant impacts at various healthcare levels, as discussed in this paper. In addition to health impact, individuals with multimorbidity face many challenges, such as navigating through the health system and community services, loss of employment, polypharmacy issues, and societal implications. Providers treating individuals with multimorbidity are often unprepared to deal with the associated complexities that go beyond the management of the diseases. Decision-makers and policy-makers are important enablers in these transformations as the complexities faced by affected individuals have implications on budget, resource management, and policies within healthcare organizations. In this paper, the experiences of dealing with multimorbidity in the ICCS outpatient clinic are presented. The care delivery model and the care team have evolved from patient, provider, and key stakeholder needs and experiences [14, 16]. Challenges related to multimorbidity have long been recognized in this facility as the majority of the patients seen have more than three chronic conditions.

Based on the exploration over the last few years, the functional health ICF model [18], whole-person model [21], and the CCM [17] have guided the service and the care team to optimally consider the multifaceted needs of this patient population. Within the health authority in Nova Scotia, a concurrent exploration is developing strategies and protocols for system-level transformations to meet the needs of multimorbidity and chronic conditions [8].

Preliminary results from the ICCS intervention are promising. We observed that over 75% of patients achieved optimal self-selected functional health as measured by the COPM. These functional outcomes are self-care, productivity (work/employment is the top-ranked self-selected functional health goal in patients), and leisure [14]. Redesigning steps and including the patient’s voice in ICCS delivery have resulted in many positive outcomes, including reduction in waiting times to care [7, 8].

The redesign experience has led to several important learning opportunities about this patient population. Individuals with multimorbidity have care needs that are unique, but many of the experienced complexities are often introduced by the complex processes within the health system. Feedback from patients during our initial review revealed experiences of these process challenges that seem to create complexities at various levels. These challenges included navigating the health system and timeliness of care received, access to comprehensive and meaningful care, and establishing goals and outcomes of care that are important and relevant to patients. Most importantly, lack of inclusion in their own care can lead to patients feeling less confident in self-management, not having their care needs met, and increased levels of dissatisfaction with the health system.

The evidence of benefit reported in this paper is primarily based on self-report and from uncontrolled before and after intervention data. Despite the promising nature of these results, in order to truly establish the benefits at the patient and health system levels, a stronger research design is planned with data on healthcare utilization, usage of emergency services, and health outcomes.

## Figures and Tables

**Table 1:** Comprehensive information for care planning collected on all Integrated Chronic Care Service (ICCS) patients.

Patient information	Source
Diagnosis information	ICCS patient intake questionnaire
Demographic information	ICCS patient intake, Stanford Chronic Disease Questionnaire
Patient self-selected functional health goals	Canadian Occupational Performance Measure
Pain and fatigue information	Stanford Chronic Disease Questionnaire
Physical and mental composite scores	12-Item Short Form Health Survey
Physical and daily activity scores	12-Item Short Form Health Survey
Confidence in self-management	Stanford self-efficacy scale
Overall perception of health	Stanford Chronic Disease Questionnaire
Nutrition, psychosocial information	Patient intake questionnaire
Patient perception and involvement in care	Patient Assessment of Chronic Illness Care Hopes and Needs Survey

**Table 2:** Patient demographics.

Demographic	*n*=456
Age, mean (SD)	45.8 (12.1)
Gender, %	
Female	87
Male	13
Education, years (SD)	12.9 (8.2)
Employment, %	
Employed	47.8
On disability	27.2
Retired, other	25.0

SD, Standard deviation.

**Table 3:** Clinical profile of Integrated Chronic Care Service (ICCS) patients.

Health characteristic (*n*=456)	Mean (SD)
>2 Conditions	98.7%
>3 Conditions	95.0%
>5 Conditions	65.6%
>8 Conditions	20.4%
Musculoskeletal conditions	65.3%
Respiratory conditions	45.3%
Mental health diagnoses	29.5%
Self-efficacy (score of 60 is optimal)	14.7 (2.1)
Daily activity (score of 15=dysfunctional)	11.4 (1.2)
Physical activity (score of 5=optimal activity)	2.6 (1.1)
Overall health (1=good health to 5=poor health)	3.9 (0.5)
Body mass index, mean (SD), kg/m^2^	25.6 (6.7)
Nutritional concerns	87.5%
Psychosocial distress	68.0%
Pain	77.6%
Fatigue	94.7%

SD, Standard deviation.

**Table 4:** Outcomes of self-selected functional health goals observed in the Integrated Chronic Care Service (ICCS) patients.

Functional goals measured with COPM	%
Self-care – self-management	95
Leisure – socializing (isolation)	94
Productivity – work, volunteering	86
Self-care – energy, fatigue management, exercise	85
Productivity – housework, meal preparation	75
Leisure – activities such as reading, yoga, walking	74
Self-care – sleep, rest	72
Self-care – coping, time management	62

COPM, Canadian Occupational Performance Measure.

## Footnotes

1 http://www.cdha.nshealth.ca/integrated-chronic-care-service-iccs

2 http://www.cdha.nshealth.ca/primary-health-care
